# Acute Vitiligo Repigmentation in the Setting of Suspected Pulmonary Sarcoidosis

**DOI:** 10.31486/toj.21.0098

**Published:** 2022

**Authors:** Brandon T. Thrash, Peter G. Pantlin, Brandy C. Mize, Colin C. Rutner, Cassie A. Shaw, Ross E. McCarron

**Affiliations:** ^1^ Louisiana State University Health Sciences Center, School of Medicine, New Orleans, LA; ^2^ Department of Internal Medicine, Louisiana State University Health Sciences Center, New Orleans, LA; ^3^ Department of Pulmonary/Critical Care, Louisiana State University Health Sciences Center, New Orleans, LA; ^4^ Department of Hospital Medicine, Louisiana State University Health Sciences Center, New Orleans, LA

**Keywords:** *Autoimmune diseases*, *sarcoidosis*, *sarcoidosis–pulmonary*, *vitiligo*

## Abstract

**Background:** Sarcoidosis is a noncaseating granulomatous disease that predominately occurs in the lungs. Vitiligo is the most common depigmentation disorder worldwide. Both diseases are autoimmune-mediated, suggesting that one could have implications for the other. However, relatively few reports have been published about patients presenting with coinciding symptoms of the 2 diseases. We report the case of a patient who presented with focal repigmentation of vitiligo with suspected pulmonary sarcoidosis.

**Case Report:** A 63-year-old female with a medical history of diffuse vitiligo reported to the emergency department with the chief complaint of right lower extremity weakness and numbness for 1 week. She reported that she had had a chronic productive cough for the prior 4 to 6 months and had unintentionally lost 50 to 60 pounds in the prior 3 months. At that time, she began to notice numerous hyperpigmented macules and patches on both forearms and her face. Chest x-ray and chest computed tomography demonstrated bilateral hilar and mediastinal lymph node enlargement with multiple bilateral pulmonary nodules. Cytology and flow cytometry were negative for evidence of B- or T-cell lymphoproliferative disorder with evidence of granulomatous inflammation.

**Conclusion:** This clinical presentation suggests a potential interplay between 2 unique disease processes. While both vitiligo and sarcoidosis share common autoimmune etiologies, little data are available about management when they coincide. This case highlights a patient with 2 seemingly distinct clinical manifestations that could yield further clinical information in the management of both diseases separately and together.

## INTRODUCTION

Sarcoidosis is a noncaseating granulomatous disease that predominately occurs in the lungs, although it can affect almost every organ system.^1^ Its severity and clinical presentation can be highly variable, and sarcoidosis can even be an incidental finding on radiographic imaging.^1^ Disease presentation tends to occur in patients between the ages of 20 to 60 years and can mimic other diseases such as lymphoproliferative disorders and granulomatous infections.^2^ No specific test is available for sarcoidosis; the diagnosis is primarily one of exclusion.^2^ Depending on disease severity, systemic corticosteroids remain the mainstay of treatment therapy and have wide-ranging results.^1^ In patients refractory to corticosteroid treatment, antimetabolites may be considered as a second-line option.^1^ However, both of these treatment modalities have significant side effect profiles that create a major hurdle in the management of disease burden in severe cases.^1^

Vitiligo is the most common depigmentation disorder worldwide, with an estimated prevalence of 1%.^3^ The pathophysiology of vitiligo is loss of melanocytes from largely uncertain mechanisms, but multifactorial genetic and environmental factors have been proposed.^3,4^ Presentation can range from localized macules to widespread hypopigmentation that can cause significant psychological stress in affected individuals.^3^ Vitiligo has 2 major forms: nonsegmental, the most common form, and segmental.^3,4^ Nonsegmental vitiligo is characterized by symmetrical and bilateral white patches, while segmental vitiligo tends to have a unilateral distribution.^3,4^

Both diseases are autoimmune-mediated, suggesting that exacerbation of one could potentially have implications for the other. However, relatively few reports have been published about patients presenting with coinciding symptoms of each disease, and most are confined to manifestations of cutaneous sarcoidosis with underlying vitiligo.^5-7^ We report the case of a patient who presented with focal repigmentation of patches in the setting of suspected symptomatic sarcoidosis with neurologic manifestations.

## CASE REPORT

A 63-year-old female with a 20-year history of diffuse vitiligo reported to the emergency department with the chief complaint of right lower extremity weakness and numbness for 1 week. She first noticed the weakness in her right foot with associated tingling on the lateral aspect of the ipsilateral ankle that progressively extended to the level of her knee. At the time of presentation, she stated that both her feet felt subjectively cold, and she was experiencing similar weakness and tingling in both hands. Two months previously, she said she felt as if something tore in her right eye, causing intermittent dark spots in her vision. An optometrist diagnosed a 1- to 2-mm hyperpigmented spot in the conjunctiva at the 10 o’clock position. During the prior 4 to 6 months, the patient reported that she had had a chronic cough with productive yellow/green sputum without blood and that she had unintentionally lost 50 to 60 pounds in the prior 3 months that she attributed to stress from the death of her husband from lung cancer. At that time, she began to notice numerous hyperpigmented macules and patches on both forearms and increasing pigmentation of her face that she further attributed to stress. She denied any recent trauma, night sweats, facial weakness, speech difficulty, bowel or bladder incontinence, uterine bleeding, or blood in her stool or urine. She denied alcohol use and illicit drug use, and she had never smoked, although she had significant secondhand smoke exposure from her husband. Her only surgical history was a cesarean section. She did not take any medications, and her family history was unremarkable.

Her vital signs were stable with oxygen saturation of 99% on room air, and her body mass index was 25.7 kg/m^2^. Physical examination was notable for slow and unsteady gait, with her right foot externally rotated and dragging during ambulation. Her finger to nose, heel to shin, gross motor strength, and sensation were all normal and intact. Her skin was diffusely hypopigmented with clusters of hyperpigmented macules and patches on bilateral forearms and face and more sparsely on her trunk and lower extremities. The remainder of her physical examination was unremarkable. Cardiac, pulmonary, and neurologic examinations were normal.

X-ray and computed tomography (CT) of the chest demonstrated bilateral hilar and mediastinal lymph node enlargement with multiple bilateral pulmonary nodules having ill-defined margins. CT head showed no abnormalities or masses. CT abdomen showed cholelithiasis without evidence of cholecystitis. Her initial blood work yielded a white blood cell count on the lower level of normal at 4.5 × 10^3^/μL (reference range, 4.5-11.0 × 10^3^/μL) with a normal differential. She had a mild iron deficiency anemia with hemoglobin of 10.9 gm/dL (reference range, 12.0-16.0 gm/dL), hematocrit of 34% (reference range, 35%-46%), and mean corpuscular volume of 79.7 fL (reference range, 80.0-100.0 fL). Total iron was decreased at 38 μg/dL (reference range, 40-160 μg/dL), and ferritin was 11.4 ng/mL (reference range, 20.0-210.0 ng/mL). Glucose was elevated at 135 mg/dL (reference range, 65-99 mg/dL), and she had an isolated elevated alkaline phosphatase of 143 U/L (reference range, 20-120 U/L). Folate, vitamin B12, prothrombin time/international normalized ratio, and activated partial thromboplastin time were all within normal ranges. To determine cardiac status, B-type natriuretic peptide, creatinine kinase, and troponin were assessed; all were within normal ranges. A nasopharyngeal swab severe acute respiratory syndrome coronavirus-2 test was negative. Lipid panel showed an elevated total cholesterol of 247 mg/dL (reference range, <200 mg/dL) and low-density lipoprotein cholesterol of 188 mg/dL (reference range, <130 mg/dL) with reduced high-density lipoprotein cholesterol of 33 mg/dL (reference range, 40-59 mg/dL).

The patient was admitted to the hospital and started on daily oral 325 mg ferrous sulfate tablets, daily 40 mg atorvastatin, and daily 81 mg aspirin for hyperlipidemia and secondary stroke prevention. Subsequent hemoglobin A1c was elevated at 6.8% (reference range, 4.7%-5.6%), and the patient received 3 separate 1-unit insulin aspart U-100 injections while admitted.

The leading differential diagnoses were malignancy with paraneoplastic syndrome given her pulmonary nodules with hilar lymphadenopathy, metastatic disease including bone, multiple sclerosis or other autoimmune etiology given her history of vitiligo, sarcoidosis despite normal calcium levels, infectious granulomatous disease, and stroke.

Urine drug screen and HIV-1/2 Ag/Ab Combo screen were negative. Magnetic resonance imaging of the brain demonstrated no evidence of acute territorial infarct, intracranial hemorrhage, or mass lesions. Magnetic resonance angiography of the head and neck was negative for stenosis, occlusion, vascular malformations, and aneurysms. Transthoracic echocardiogram with bubble study showed normal systolic and diastolic function and no septal defects. T-SPOT.*TB* (Oxford Immunotec Ltd) tuberculosis test was negative, hepatitis C antibody screen was negative, and inflammatory markers were within normal limits. Complement C3 and C4 levels were within normal limits, antinuclear antibody screen was negative, and cytoplasmic antineutrophil cytoplasmic autoantibodies were <1:20 (reference range, <1:20). Blood and urine cultures yielded no growth. Of note, a daily complete blood count during admission demonstrated leukopenia with white blood cell counts of 4.4 × 10^3^/μL followed by 4.3 × 10^3^/μL. The patient refused a skin punch biopsy for further dermatologic workup.

She was discharged home 2 days after admission on 81 mg aspirin daily, 40 mg atorvastatin daily, and 325 mg oral iron sulfate daily. She declined starting metformin and was counseled on lifestyle modifications for newly diagnosed diabetes mellitus. Her leading diagnoses at that time were a lymphoproliferative disorder, metastatic disease, and infectious vs noninfectious granulomatous disease. She was scheduled for an outpatient endobronchial ultrasound-guided transbronchial needle aspiration (EBUS-TBNA) with biopsies 3 days after her discharge for further pulmonary evaluation.

Cytology and flow cytometry from the EBUS-TBNA were negative for evidence of B- or T-cell lymphoproliferative disorder. Some evidence of granulomatous inflammation at stations 7 and 11 suggested possible granulomatous disorder. Fungal and acid-fast bacteria cultures were negative. The patient was scheduled for outpatient management with pulmonary function tests, repeat chest CT, referral to hematology/oncology for possible bone marrow biopsy, and referral to gastroenterology for colonoscopy.

The consultation with hematology/oncology after discharge was unremarkable, with a continued suspected diagnosis of a chronic inflammatory disorder such as sarcoidosis based on EBUS-TBNA results. Repeat CT of the chest 3 months postdischarge demonstrated overall improved bilateral areas of hilar nodularity, despite lack of treatment. Given this generalized improvement without treatment, a lymphoproliferative disorder became less likely and further suggested granulomatous disease, such as sarcoidosis. Of note, a few new nodules were noted from previous imaging, while others were no longer evident. Esophagogastroduodenoscopy with colonoscopy yielded a benign colonic polyp and generalized gastritis with multiple gastric ulcers positive for *Helicobacter pylori.* The patient was prescribed 40 mg pantoprazole sodium twice daily for 2 weeks followed by once daily for 6 weeks and 3 bismuth-metronidazole-tetracycline 40-125-125 mg capsules 4 times daily for 2 weeks. Periodic chest surveillance was recommended for hilar nodularity.

## DISCUSSION

This case demonstrates the potential simultaneous clinical presentation of 2 disorders. The patient presented with a chief complaint of acute onset lower extremity weakness and numbness with bilateral hilar lymphadenopathy incidentally found on chest imaging. The patient also had a chronic productive cough and macules of spontaneous repigmentation of her long-standing vitiligo. Both of these disease processes are autoimmune-mediated, yet little evidence has been published of the 2 occurring symptomatically at the same time outside of cases confined to dermatologic lesions or induced after systemic therapy.^5-10^ To our knowledge, no cases of simultaneous vitiligo reactions and sarcoidosis with pulmonary manifestations have been reported.

The pathophysiology of vitiligo is complex and multifactorial with a dynamic interplay between genetics and environment.^11^ Genetic studies have identified many predisposing variants that code for components of the innate and adaptive immune system.^11^ Research suggests that these variants could impair the ability of melanocytes to manage oxidative stress and the exposure to certain environmental factors; however, this process is not fully understood.^11^ Ultimately, melanocytes are destroyed by cytotoxic CD8+ T cells through local chemokine signaling within the epidermis, resulting in the characteristic hypopigmentation.^11^

Sarcoidosis is a multisystem disease of unknown etiology characterized by the formation of nonnecrotizing epithelioid granulomas in a lymphatic pattern around bronchovascular structures.^12^ Histologically, the granulomas contain epithelioid cells, giant cells, and CD4+ T cells in their center and CD8+ T cells and B cells at their periphery.^12^ Therefore, while each disease has a distinct pathogenesis, they share common autoimmune cell–mediated pathologies, particularly within the adaptive immune system.

An important factor in this case is clinical management: how to adequately treat each disorder individually with consideration of the other. The mainstay of treatment in vitiligo can be highly variable and patient specific. Thorough repigmentation is often difficult to achieve, and most modalities are aimed at preventing disease progression.^4^ Current management usually includes topical corticosteroids and immunomodulators.^4^ Interventions such as narrow-band ultraviolet B and pigment cell transplantation^4^ can be used in advanced cases but are less widely available and likely cost-prohibitive. On the other hand, systemic corticosteroids are used to treat sarcoidosis for the majority of patients.^1^ Steroid treatment can be used for an extensive duration; the side effect profile is commonly the limiting factor in management.^1^ In refractory cases, antimetabolites may be used as a second option.^1^ Thus, considering the common autoimmune pathophysiology and the interactions between treatment modalities of both vitiligo and sarcoidosis, co-management of each could have potentially beneficial or adverse reactions on the other.

However, a lymphoproliferative disorder cannot be fully ruled out. While EBUS-TBNA is commonly a first-line approach to diagnosing hilar lymphadenopathy, studies have shown EBUS-TBNA to have an overall sensitivity for de novo diagnosis ranging from 78% to 92%.^13,14^ Because of limitations in small-volume needle biopsies, negative samples obtained with high suspicion for lymphoproliferative disorders warrant further investigation.^14^ Thus, while the samples obtained from this patient were negative for lymphoproliferative disorder, diagnostic testing such as a bone marrow biopsy should be done before a definitive conclusion can be reached. Further, a skin biopsy of a lesion would need to be obtained before cutaneous sarcoidosis could be effectively ruled out. However, the macules were flat and compatible with the patient's natural skin pigmentation, and there was little clinical suspicion for etiologies other than spontaneous vitiligo repigmentation. Nonetheless, small hypopigmented patches of cutaneous sarcoidosis masquerading as vitiligo have been reported.^9,10^ These cases appear to be distinct from our patient as the lesions were small annular patches or macules without evidence or history of diffuse hypopigmentation or pulmonary symptoms.^9,10^ Despite these distinctions, cutaneous sarcoidosis has been shown to present as hypopigmented lesions, which cannot be truly rejected in our case without biopsy confirmation.^9,10,15^ Also, this presentation is different than other cases of cutaneous sarcoidosis with underlying vitiligo in which the lesions typically appear as pink and nodular.^5-7^

## CONCLUSION

This case suggests a potential overlap and interplay between 2 unique disease processes. While both vitiligo and sarcoidosis share common autoimmune etiologies, little data are available about the management of the 2 when their symptomatic presentations coincide. More investigation and clinical evaluation are needed to determine the appropriate treatment algorithm in this setting, as well as how management of one disorder could impact the other. This case highlights a novel case of 2 seemingly distinct clinical manifestations that could yield further clinical information in the management of both diseases separately and together.

## Notes

*This article meets the Accreditation Council for Graduate Medical Education and the American Board of Medical Specialties Maintenance of Certification competencies for Patient Care and Medical Knowledge.*
